# Hypoxia and Inactivity Related Physiological Changes (Constipation, Inflammation) Are Not Reflected at the Level of Gut Metabolites and Butyrate Producing Microbial Community: The PlanHab Study

**DOI:** 10.3389/fphys.2017.00250

**Published:** 2017-05-04

**Authors:** Robert Šket, Nicole Treichel, Tadej Debevec, Ola Eiken, Igor Mekjavic, Michael Schloter, Marius Vital, Jenna Chandler, James M. Tiedje, Boštjan Murovec, Zala Prevoršek, Blaž Stres

**Affiliations:** ^1^Department of Animal Science, Biotechnical Faculty, University of LjubljanaLjubljana, Slovenia; ^2^Research Unit for Comparative Microbiome Analysis, Helmholtz Zentrum München - German Research Center for Environmental HealthNeuherberg, Germany; ^3^Department of Automation, Biocybernetics and Robotics, Jozef Stefan InstituteLjubljana, Slovenia; ^4^Department of Environmental Physiology, Swedish Aerospace Physiology Centre, Royal Institute of TechnologyStockholm, Sweden; ^5^Center for Microbial Ecology, Michigan State UniversityEast Lansing, MI, USA; ^6^Laboratory for Artificial Sight and Automation, Faculty of Electrical Sciences, University of LjubljanaLjubljana, Slovenia; ^7^Center for Clinical Neurophysiology, Faculty of Medicine, University of LjubljanaLjubljana, Slovenia

**Keywords:** human intestinal microbiome, hypoxia, inactivity, inflammation, constipation, gut metabolites, butyrate producing community, noncommunicable diseases

## Abstract

We explored the assembly of intestinal microbiota in healthy male participants during the run-in (5 day) and experimental phases [21-day normoxic bed rest (NBR), hypoxic bedrest (HBR)], and hypoxic ambulation (HAmb) in a strictly controlled laboratory environment, balanced fluid, and dietary intakes, controlled circadian rhythm, microbial ambiental burden, and 24/7 medical surveillance. The fraction of inspired O_2_ (F_i_O_2_) and partial pressure of inspired O_2_ (P_i_O_2_) were 0.209 and 133.1 ± 0.3 mmHg for NBR and 0.141 ± 0.004 and 90.0 ± 0.4 mmHg for both hypoxic variants (HBR and HAmb; ~4,000 m simulated altitude), respectively. A number of parameters linked to intestinal transit spanning Bristol Stool Scale, defecation rates, zonulin, α_1_-antitrypsin, eosinophil derived neurotoxin, bile acids, reducing sugars, short chain fatty acids, total soluble organic carbon, water content, diet composition, and food intake were measured (167 variables). The abundance, structure, and diversity of butyrate producing microbial community were assessed using the two primary bacterial butyrate synthesis pathways, butyryl-CoA: acetate CoA-transferase (*but*) and butyrate kinase (*buk*) genes. Inactivity negatively affected fecal consistency and in combination with hypoxia aggravated the state of gut inflammation (*p* < 0.05). In contrast, gut permeability, various metabolic markers, the structure, diversity, and abundance of butyrate producing microbial community were not significantly affected. Rearrangements in the butyrate producing microbial community structure were explained by experimental setup (13.4%), experimentally structured metabolites (12.8%), and gut metabolite-immunological markers (11.9%), with 61.9% remaining unexplained. Many of the measured parameters were found to be correlated and were hence omitted from further analyses. The observed progressive increase in two immunological intestinal markers suggested that the transition from healthy physiological state toward the developed symptoms of low magnitude obesity-related syndromes was primarily driven by the onset of inactivity (lack of exercise in NBR) that were exacerbated by systemic hypoxia (HBR) and significantly alleviated by exercise, despite hypoxia (HAmb). Butyrate producing community in colon exhibited apparent resilience toward short-term modifications in host exercise or hypoxia. Progressive constipation (decreased intestinal motility) and increased local inflammation marker suggest that changes in microbial colonization and metabolism were taking place at the location of small intestine.

## Introduction

Many studies investigating host–microbiota interactions have demonstrated that numerous parameters drive the structural and functional changes of the human gut microbiome. Its critical role in maintaining gut homeostasis and host health has been confirmed in a number of studies comparing various end-point groups with significantly different symptoms (Clarke et al., 2014; Schloss et al., 2014) and also in investigations employing longitudinal approach over time (Vital et al., 2013; David et al., 2014a,b; Thaiss et al., 2014; Figure S1).

In this respect, the role of exercise as a modulator of the gut microbiome has received attention as a possibility of reducing the risk for several metabolic, inflammatory and neoplastic diseases for inactive individuals (Pham et al., 2012; van Dijk et al., 2012; Egan and Zierath, 2013; Cronin et al., 2016). Various levels of physical exercise were recently linked to modifications of the microbiota in test-participants (Clarke et al., 2014), increased vagal-nerve tone at rest (Cronin et al., 2016), gene expression of transport proteins (Jayewardene et al., 2016) and exercise related immunological responses (Bermon et al., 2015; Ringseis et al., 2015). Furthermore, Clarke et al. (2014) clearly outlined the close link between the triad of diet, metabolism and exercise as increased exercise and dietary extremes strongly influenced the microbial diversity in professional athletes (Figure S2). However, most studies so far have focused on the magnitude of symptoms as a result of prolonged inactivity and the effects of the restoration of physical activities in an unfit population.

From a physiological perspective, inactivity was shown to result in tissue hypoxia through reduced capillary oxyhemoglobin saturation, changes in muscle composition, modified expression of genes, and cellular metabolism and release of pro-inflammatory cytokines (Bermon et al., 2015; Ringseis et al., 2015; Liao et al., 2016). On the other hand, adaptation to hypoxia results in coordinated cascade-like down-regulation of metabolic demands and supply to prevent a mismatch in ATP utilization and production in cell tissues (Wheaton and Chandel, 2011). Hypoxia and inflammation therefore appear to be interdependently related as many publications implicated inflammation during hypoxic conditions to be related to a wide range of human diseases (Bartels et al., 2013). For instance, life-long inactivity and obesity-related disorders were clearly associated to differences in human intestinal microbiome between healthy and affected groups. However, the multifaceted relationship between the decreased physical exercise (i.e., inactivity), human physiology, intestinal microbiota, accompanying intestinal metabolites, and diet have not been explored during acute cessation of exercise in healthy test participants.

To improve our understanding regarding the pathophysiological consequences of inactivity and hypoxia, this study took advantage of the PlanHab project experimental setup (Debevec et al., 2014; Simpson et al., 2016) designed to investigate in controlled manner the combined effects of prolonged (21 day) inactivity/unloading and hypoxia on healthy-volunteers. The experiment was conducted according to European Space Agency (ESA) and NASA core bedrest data collection SOP (Standardization of bed rest study conditions 1.5, August 2009), and included controlled daily water and nutrition intake next to controlled circadian rhythm, microbial ambiental burden and 24/7 medical surveillance (Figure S1).

Intestinal microbiota of healthy male test participants was explored during the run-in (5 day) and consecutive experimental phases [21-day normoxic bed rest (NBR), hypoxic bedrest (HBR) and hypoxic ambulation (HAmb)]. A number of parameters linked to intestinal transit were measured: (i) feces consistency (Bristol Stool Scale) and defecation rates; (ii) four immunological markers targeting colonocite permeability, mucus integrity, on-site inflammation and lipid absorption/biocidal effects; (iii) description of fecal environment (pH) and measurements of concentration of C1–C6 short chain fatty acids (SCFA), total SCFA, reducing sugars, bile acids (BA), total soluble organic carbon (TSOC), water content; (iv) abundance, diversity, and structure of butyrate producing microbial community through targeting the two primary bacterial butyrate synthesis pathways, butyryl-CoA: acetate CoA-transferase (*but*) and butyrate kinase (*buk*) genes.

We hypothesized that reduced physical activity would provoke notable changes in the nutrient status of the intestinal tract, increase gut permeability and inflammation, and change the functional microbial communities relative to the initial active stages. Systemic hypoxia was expected to act as an additional confounding factor aggravating the observed changes within the 21 day time frame, whereas ambulation in hypoxia was anticipated to at least partially ameliorate these effects due to increased physical activity levels.

## Materials and methods

In the frame of the present sub-study within the PlanHab project (registration number NCT02637921 at www.ClinicalTrails.gov) the dynamics and diversity of fecal bacterial communities were studied as in response of reduced activity and hypoxia and linked to fecal metabolites (SCFA, reducing sugars) as well as immunological markers of the gut barrier integrity [zonulin, α_1_-antitrypsin (A1AT), eosinophil-derived neurotoxin (EDN) and bile acids (BA)]. The detailed outline of the PlanHab project is given elsewhere (Debevec et al., 2014, 2016; Keramidas et al., 2016; Rittweger et al., 2016; Rullman et al., 2016; Simpson et al., 2016) but is also summarized below for the convenience of the reader.

### Study design and setting

The PlanHab study was performed between October 2012 and December 2013 at the hypoxic facility at the Olympic Sport Center Planica in Rateče, Slovenia, located at 940 m of altitude. The study was conducted according to the ESA plan for standardization of bed rest studies (Debevec et al., 2014). For this study, each participant underwent 5 days of baseline data collection (BDC) during which participants were ambulant, 21 intervention days (normoxic bed rest, hypoxic bed rest, or hypoxic ambulation), and 5 days of medical follow-up. For BDC, operational days are indicated by “BDC-n,” meaning n days before the onset of bed rest.

In particular, the participants underwent the following three protocols in a randomized and counterbalanced manner: (1) normobaric normoxic bed rest [NBR; fraction of inspired O_2_ (F_i_O_2_) = 0.209; partial pressure of inspired O_2_ (P_i_O_2_) = 133.1 ± 0.3 mmHg]; (2) normobaric hypoxic ambulatory confinement (HAmb; F_i_O_2_ = 0.141 ± 0.004; P_i_O_2_ = 90.0 ± 0.4 mmHg; ~4,000 m simulated altitude); and (3) normobaric hypoxic bed rest (HBR; F_i_O_2_ = 0.141 ± 0.004; P_i_O_2_ = 90.0 ± 0.4 mmHg; ~4,000 m simulated altitude).

Prior to commencement, the study had received approval by the National Committee for Medical Ethics at the Ministry of Health of the Republic of Slovenia. In addition to the present sub-study, other published sub-studies or in preparation focused upon cardiorespiratory, muscular (Debevec et al., 2014), immunological (Keramidas et al., 2016), psycho-neuroendocrine responses (Keramidas et al., 2016; Strewe et al., 2017) next to appetite-regulation (Debevec et al., 2016), skeletal muscle miRNA expression (Rullman et al., 2016) and mineral metabolism (Rittweger et al., 2016) within the same participants.

### Test participants

Inclusion and exclusion criteria for the study were based on the European Space Agency's standardization plan for bed rest studies (ESA, 2009; Debevec et al., 2014, 2016) and aimed at selecting participants who could safely undergo bed rest (e.g., exclusion of people with osteoporosis, with blood clotting disorders, history of deep vein embolism, lower back pain, and respiratory disorders). In addition, participants assessed were also excluded from this study if they had been exposed to altitudes ≥ 2,000 m, 2 months prior to study commencement. Eleven healthy men with a mean age (± SD) of 26.4 ± 4.6 years, a height of 179.6 ± 4.7 cm, a mass of 75.9 ± 9.3 kg and a body mass index of 23.5 ± 2.7 kg/m^2^ finished all interventions. The participants were all physically active male engaged in recreational sports activities for 2–4 h per week. Their habitual daily physical activity was assessed via SOP questionnaire prior to the inclusion in the study (Debevec et al., 2014).

### Bed rest and environmental protocol

Environmental conditions were controlled (ambient temperature: 24.4 ± 0.7°C, relative humidity: 53.5 ± 5.4%) or assessed (ambient pressure: 91.2 ± 5.3 kPa). The light:dark cycle was set to 16:8 h, with bed time between 23:00 and 7:00.

SpO_2_ was measured daily at 7:00 AM with a finger oximetry device 3100 WristOx device (Nonin Medicals, Minnesota, USA) and also as part of a sleep polysomnographic study. To the latter purpose, full ambulatory polysomnography (Nicolet One, Viasys, Healthcare, Neurocare, Madison, WI, USA) was performed using standard setups (Debevec et al., 2016).

During the bed rest phase of the NBR and HBR campaigns, the participants were confined to bed in the horizontal position for 24 h/day (no negative tilt), and all activities of daily life took place in bed. One pillow was allowed for head support. Showers were taken on a specific gurney, and hospital equipment was used for bowel movements and urine collection. Compliance with the bed rest protocol was ascertained by supervision through members of staff and through closed-circuit television. No physical activity was allowed during NBR and HBR campaigns, except for changing position between supine, prone and lateral. During HAmb, participants were confined to the hypoxic area, but remained ambulatory and out of bed during the day. In order to replicate their habitual bone loading during the confinement periods, participants performed low-level physical activity in two 30-min bouts per day. The exercise was rotated using stepping, cycling and dancing. Telemetric heart rate monitoring was used to achieve the targeted heart rate (123 ± 4 beats/min) during the exercises, which was set to the heart rate observed at 50% of hypoxia specific peak power output assessed at the onset of the intervention (for more details see Debevec et al., 2014).

During HBR and HAmb campaigns, normobaric hypoxia was generated within the hypoxic area by a vacuum pressure swing adsorption system (b-Cat, Tiel, the Netherlands). Regulation of O_2_ concentration was actuated within each room at 15-min intervals. For safety reasons, participants carried portable O_2_ sensors (Rae PGM-1100, California, USA) at all times (Debevec et al., 2014, 2016).

### Diet

As reported earlier (Debevec et al., 2014; Simpson et al., 2016), the participants were provided with an individually tailored, standardized, and controlled diet throughout the intervention. Energy requirements were assessed with the Harris-Benedict method, and correction factors of 1.4 and 1.2 were used to account for activity levels in the ambulatory phases and the bed rest phases, respectively. In addition to a controlled intake of fat (30%) and protein (1.2 g per kg body mass), sodium intake was set to 3,500 mg per day. Participants were supplemented with 1,000 IU vitamin D3 per day. Fluid intake was *ad libitum*, but participants were encouraged to drink at least 28.5 mL per kg per day. Importantly, menu plans were cycled in the same way for each participant across the three experimental conditions, adjusting the quantity according to activity factors above.

### Sampling

Fecal samples were collected at the time of defecation in aseptical containers to prevent cross-contamination. Longitudinal sampling was performed for 9 participants with sampling at days −5 and −1 before the onset of experiments and days 3, 10, 18 and 21 of treatment. Altogether 54 samples were collected and immediately frozen at −20°C. Samples were subsequently aliquoted in frozen state for analyses of metabolites, immunological markers and DNA extraction preceding subsequent molecular analyses.

### Characterization of fecal samples: bristol stool scale, metabolites, pH, MWI

Stool samples were characterized for a number of parameters (Table S1) as following: Bristol stool scale (BSS) (Heaton et al., 1992; Degen and Phillips, 1996), water content, pH (Stres et al., 2013), TSOC, SCFA (Kolbl et al., 2014), reducing sugars content [4-hydroxybenzoic acid hydrazide (PAHBAH)] (Lever, 1977), molecular weight (MW) and complexity of dissolved organic carbon (DOM) using molecular weight indices (MWI) (Twardowski et al., 2004; Helms et al., 2008).

BSS was used to map the stool consistency into seven consistency categories. The highest scores in general correspond to loose stools and fast intestinal transit, whereas lowest scores correspond to constipation, hard stools, and longer colon transit times. In addition, BSS as stool consistency measure was recently implicated as an important environmental variable influencing intestinal microbiota composition (Tigchelaar et al., 2015; Gilbert and Alverdy, 2016; Vandeputte et al., 2016). Frequency of defecation was calculated for each week in experiment using all available data points.

TSOC as a measure of the total content of organic carbon concentration in the fecal sample was measured in a miniaturized 96-well format assay using Agilent GC 2.5 mL vials and teflon septa caps. For calculations of TSOC calibration curves prepared from either 1 g/L of 60°C dried glucose or humic acid (Sigma-Aldrich, Germany) were used to assess the extent of carbon complexity. Dual wavelength spectrophotometry (BIOTEK ELx808, Bio Tek Instruments, USA) was used to acquire two sets of data through monitoring reagent consumption (420 nm) and product formation (595 nm).

The extraction of C1-C6 SCFA (Acetic, Propionic, iso-Butyric, n-Butyric, iso-Valeric, n-Valeric, n-Capric acid) was conducted as described before (Kolbl et al., 2014, 2016) using a gas chromatograph (Agilent 6890, Agilent, Germany) equipped with a capillary column (Agilent J & W GC columns DB-FFAP, 30 m × 0.530 mm × 1 μm layer of stationary phase). The injector and detector temperature (FIS-flame ionizing) were 200 and 300°C, respectively. Initial oven temperature was 70°C with residence time of 1 min, which was ramped for 20°C/min to 120°C and further 10°C/min to a final oven temperature of 200°C with a residence time of 3 min. Carrier gas (mobile phase) was Helium with flow rates of 5 mL/min and nitrogen with flow rates of 25 mL/min; the detector gas was hydrogen with flow 30 mL/min and synthetic air with flow 400 mL/min. Among SCFA, butyrate is considered as one of the most important metabolites as it serves as the major energy source for colonocytes, has anti-inflammatory properties, and regulates gene expression, differentiation, and apoptosis in host cells (Vital et al., 2014).

Reducing sugar content was determined in microtiter plate format (Stres et al., 2013). Reagent was prepared immediately before analyses (Lever, 1977). After 10 min incubation at 95°C 200 μl aliquots were transferred to microtiter plates and readings collected at 490 nm. A calibration curve was prepared from serially diluted 1 g/L glucose (Sigma).

The relationship between the DOM MW was characterized using spectrophotometric measurements according to Helms et al. (2008) and Twardowski et al. (2004) generating a number of MWI. Fecal samples were centrifuged at 13,000 × g and supernatants were diluted 1:50. 200 μl of each supernatant was transferred in a well of a 96 well transparent microtiter plate (Greiner UV-Star®, Greiner, Germany); spectra from 250 to 800 nm with 5 nm step were measured using a photometer (Multiscan^®;^ Spectrum #1,500; Thermo Fisher Scientific Inc., USA). Absorbance measurements were transformed to Napierian absorption coefficient (a) using equation: *a* = 2.303^*^ A/l, where l represents the length of the beam = 2/3 cm. From the absorption spectra various parameters were calculated according to Helms et al. (2008) to characterize chromogenic dissolved organic matter. These parameters included the linear regression of natural logarithm of the transformed absorption coefficient from 275 to 295 nm and from 350 to 400 nm, respectively, as well as the ratio of these slopes (SR; 275–295 nm slope: 350–400 nm slope). In order to map the DOM MW the following polymers were used: salicylic acid (one aromatic ring), methyl red (two aromatic rings), bromocresol green and resazurin (three aromatic rings), brilliant blue (five aromatic rings), congo red and trypan blue (six aromatic rings).

### Gut barrier integrity, permeability, and inflammation

In addition to gut metabolites and physicochemical parameters, four immunological markers [zonulin, α_1_-antitrypsin (A1AT), eosinophil-derived neurotoxin (EDN), bile acids (BA)] measuring colonocite permeability, mucus integrity, on-site inflammation and lipid absorption/biocidal effects, respectively, were determined in fecal samples using four distinct ELISA tests according to manufacturer's instructions (Immundiagnostik AG, Bensheim, Germany; Table S2).

### DNA extraction

Total genomic DNA (gDNA) was extracted from aliquoted fecal samples using MOBIO Power Fecal DNA extraction Kit (MOBIO; California, USA) according to the manufacturer's instructions. In essence, triplicate homogenized subsamples of 0.25 g fresh weight were adopted for extraction. Concentration and purity (A230, A260, and A280) were determined spectrophotometrically using either NanoVue (Germany) or NanoDrop (USA). Additional external DNA standards were used for verification of quantification. The resulting DNA extracts were stored in 25 μl aliquots at −20°C until their inclusion in molecular analyses.

### Amplicon sequencing and quantification of butyrate producing bacterial communities

Abundance and diversity of intestinal butyrate-producing bacterial community was analyzed targeting the two primary bacterial butyrate synthesis pathways, butyryl-CoA: acetate CoA-transferase (*but*) and butyrate kinase (*buk*) as described in Vital et al. (2015) applying the primers from Vital et al. (2013). On average (± SD) 50,816 ± 9,855 and 73,183 ± 10,169 sequences per sample were retrieved for *but* and *buk*, respectively. Raw reads were merged (Cole et al., 2014) and subjected to FrameBot (Wang et al., 2013), where a manually curated database based on the Functional Gene Database/Repository (Fish et al., 2013) served as reference source as described before (Vital et al., 2015). All amplicons displaying at least 80% protein homology to a true *but/buk* reference were aligned to respective Hidden Markov Models (Fish et al., 2013) and subjected to complete-linkage clustering (95% protein identity; Cole et al., 2014).

Abundance estimations based on band intensity after PCR amplification was performed according to Vital et al. (2015). Abundances were estimated by categorizing band intensities of PCR products into six distinct brightness groups (0–5) that were related to standard curves established with reference genomes. qPCR on selected taxa (*Roseburia/E. rectale* and *F. prausnitzii*) was performed according to Vital et al. (2013).

### Statistical analyses

A number of ecological indices were used to assess α-diversity of samples: Taxa_S, Individuals, Dominance_D, Simpson_1-D, Shannon_H, Evenness_e^∧^H/S, Brillouin, Menhinick, Margalef, Equitability_J, Fisher_α, Berger-Parker, Chao-1 as implemented in mothur (Schloss et al., 2009). One-way NP-MANOVA was used to determine significant differences between samples and experimental variants relative to BDC.

Multiple-group comparisons were performed using Benjamini-Hochberg false discovery rate (FDR) multiple test correction (Benjamini and Hochberg, 1995; Benjamini and Yekutieli, 2001). Based on presence/absence pattern and abundance of operational taxonomic units (OTUs) in each sample distance matrices were calculated using Bray-Curtis similarity and graphically illustrating each community sample as a point on a two-dimensional graph using non-metric multidimensional scaling (NM-MDS) as implemented in the R package vegan version 2.2-0 (Oksanen et al., 2014).

Variation partitioning of variables (Table S1) distributed into (i) metabolites and immune markers (*n* = 45), (ii) experimental design (*n* = 12), and (iii) diet (*n* = 110) was conducted in R (Legendre and Legendre, 2012). A step-down procedure was adopted for each group of variables to test for univariate association of variables with the structure of microbial communities and co-correlated variables were removed from further analyses. This yielded a smaller set of variables in three explanatory matrices significantly associated with microbial communities (*n*_permutations_ = 5,000; *p* < 0.05). Variation partitioning was used to determine most important factors recorded in metadata associated with the dispersion of butyrate producing community OTUs and the extent of explained variation in the structure of communities.

### Storage of sequencing data

Acquired sequencing reads were deposited and are freely accessible on Metagenomics RAST (MG-RAST) database server (http://metagenomics.anl.gov/; Meyer et al., 2008) under project accession number mgp79441 (http://metagenomics.anl.gov/linkin.cgi?project=mgp79441).

## Results

### Inactivity affects fecal consistency

**Figure 1:**
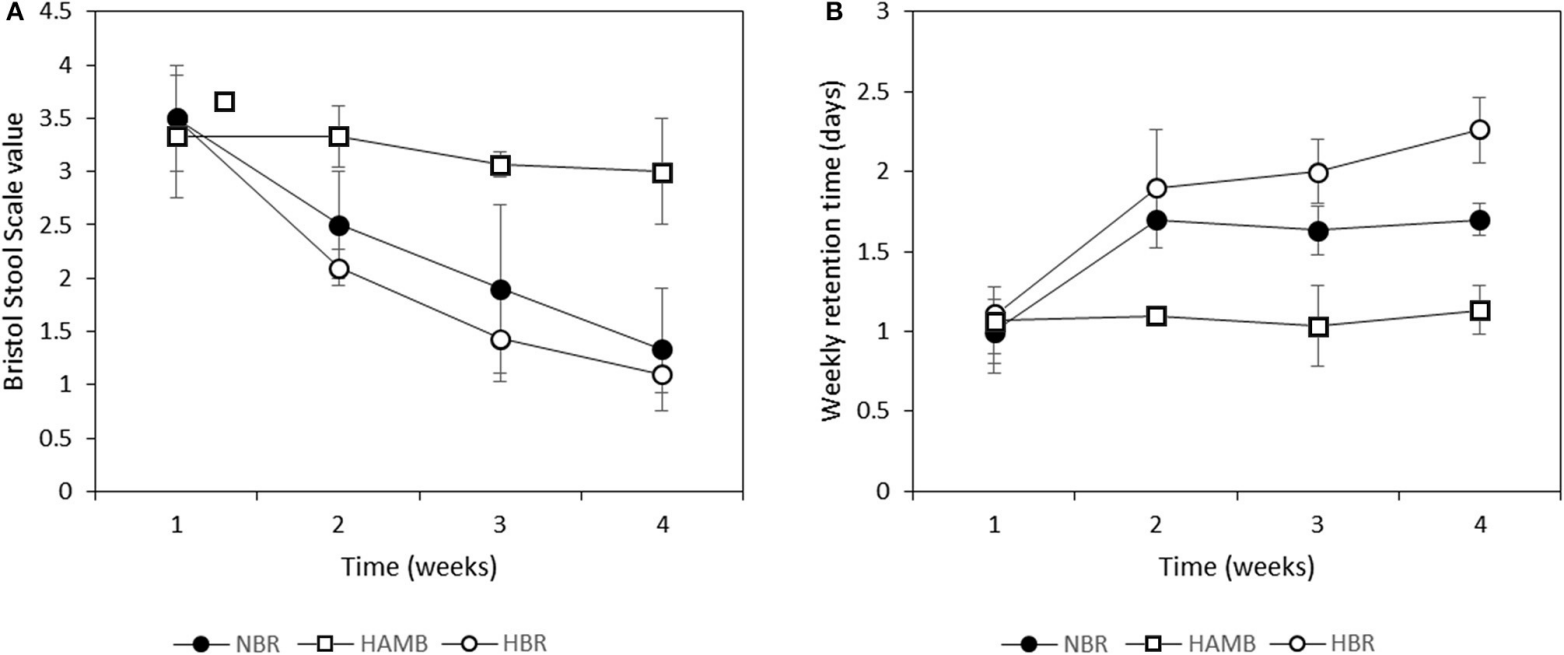
**Changes in Bristol stool scale values (A)** and retention time (as time between particular defecations) **(B)** during run-in (week 1) and subsequent 3-week experimental phase.

Significant changes were observed in consistency of fecal material relative to BDC within and between variants (Figure 1A). A pronounced decrease in fecal BSS toward constipation was observed for both bedrest variants (Figure 1B). Faster and more pronounced progression toward constipation was observed in the HBR than in the NBR participants, although this difference was not significant (*p* = 0.14). In addition, the apparent weekly retention time (as time between defecation events) was significantly higher in NBR and HBR in comparison to HAmb (Figure 1B). Relatively limited extent of daily physical activity in HAmb provided an effective protection toward constipation when the same type of controlled diet was used, despite hypoxia.

### Inactivity and hypoxia aggravate the state of gut inflammation but not permeability

**Figure 2:**
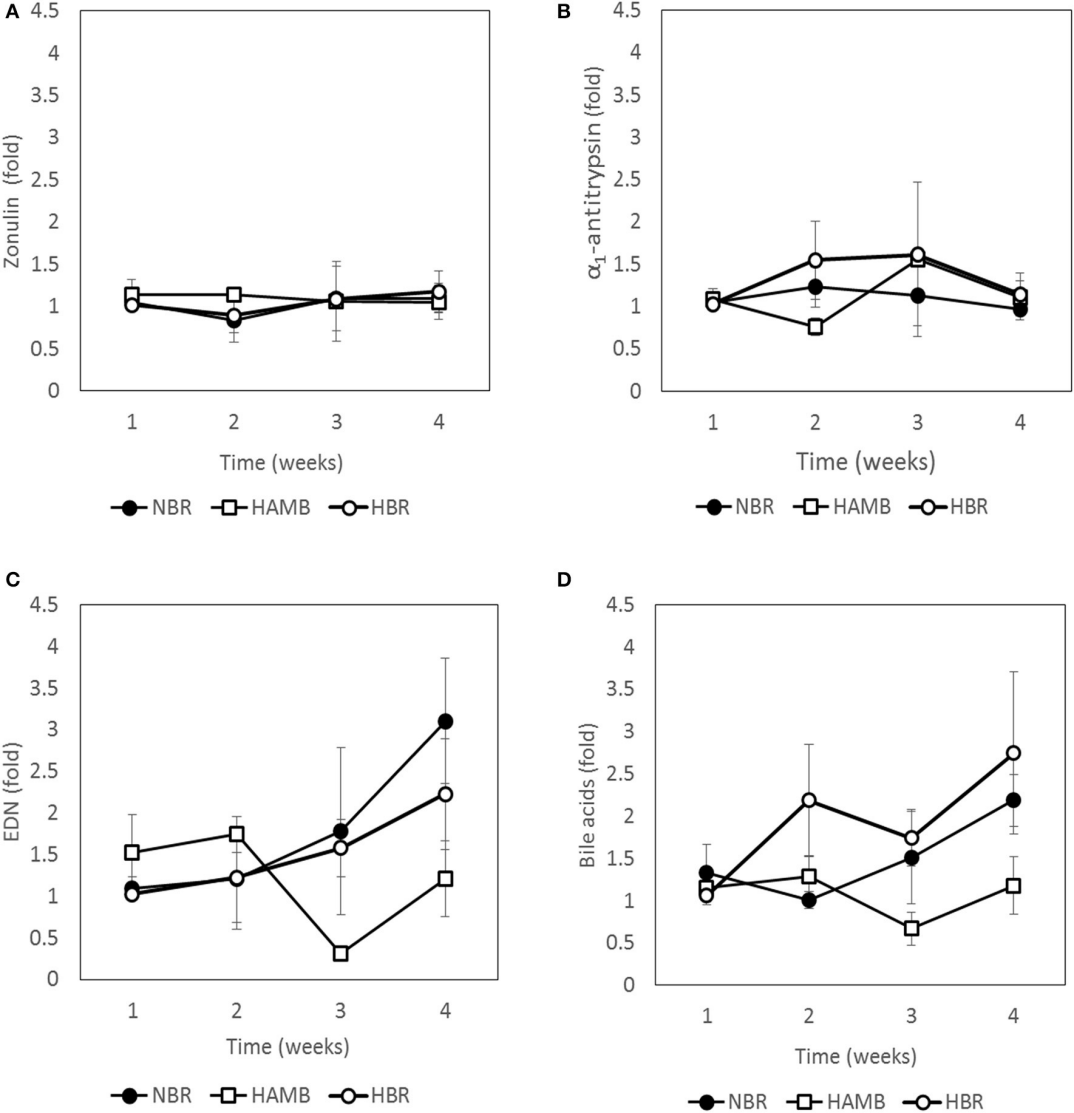
**Changes in immunological markers present in fecal samples: zonulin (A)**, α_1_-antitrypsin **(B)**, eosinophil-derived neurotoxin (EDN) **(C)** and bile acids **(D)** during run-in (week 1) and subsequent 3-week experimental phase.

Four immunological markers, mapping the tight junction leakage (zonulin), mucosal integrity (A1AT), on site inflammation (EDN), and pro-inflammatory cytokine pathways/lipid metabolism (BA) were determined over time for all participants (Figure 2). The two markers describing gut leakage, zonulin (chemical flow through tight junctions into blood stream), and A1AT (mucosal integrity and mucosal permeability in direction from the host toward intestinal lumen), were not significantly different between experimental variants over time, suggesting that a healthy epithelium and mucosal integrity were maintained over the course of the experimental period, irrespective of inactivity or hypoxia.

In contrast, clear shifts after exposure were detected for EDN and BA (Figure 2). EDN was highest in HBR, followed by NBR, with limited fluctuations in HAmb. BA increased most in NBR, but was not significantly higher than in HBR, whereas in fecal samples from participants of the HAmb treatment BA was not significantly increased until the end of experiments despite hypoxia. The results show that inactivity clearly increased fecal BA content, which is an indicator for increased local inflammation in the intestinal tract and higher cytotoxic activity toward microbiota, whereas hypoxia had no measurable negative effect under the chosen experimental setup.

### Inactivity and hypoxia did not affect gut metabolic markers

**Figure 3:**
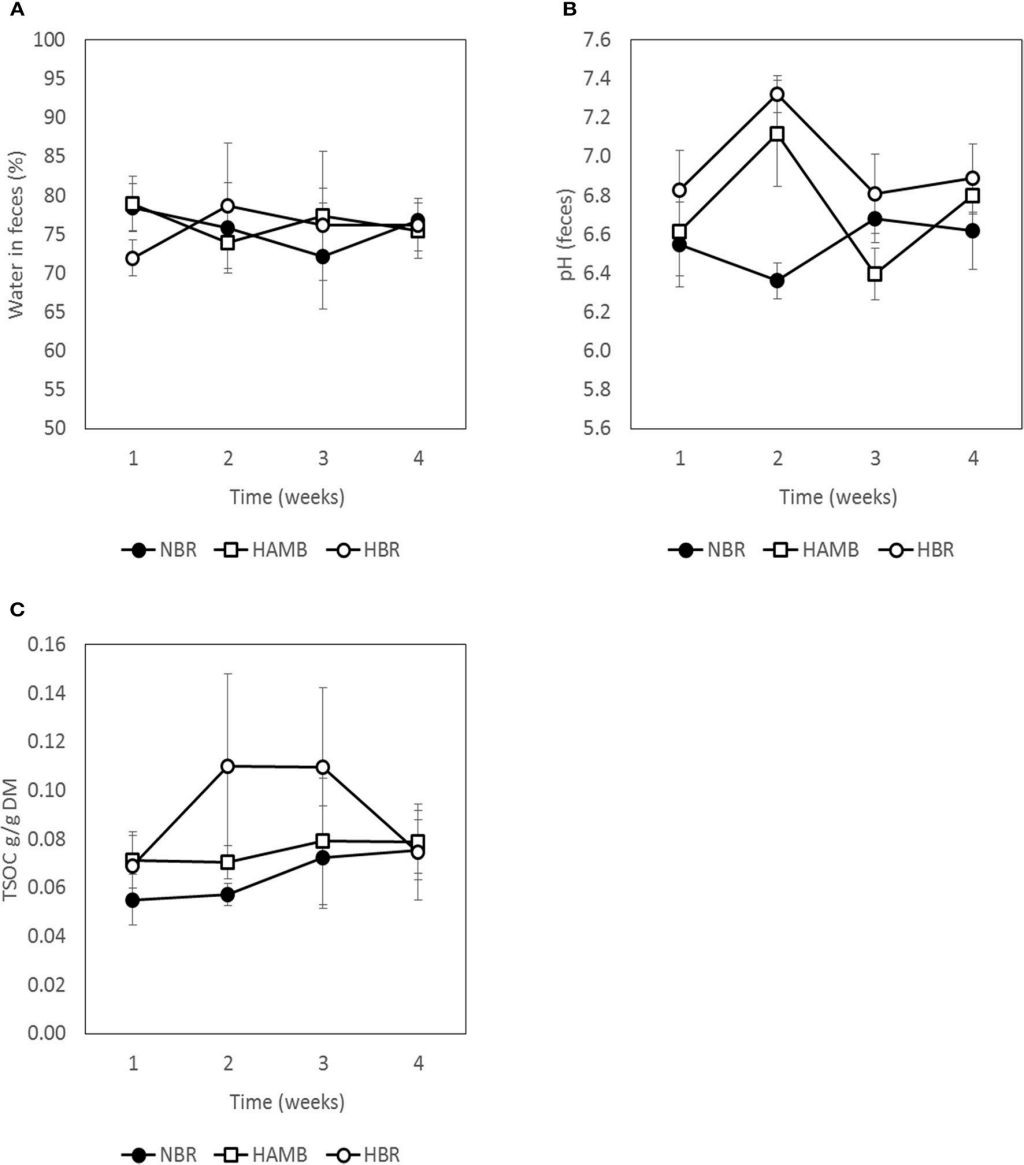
**Variation in water content (A)**, pH **(B)**, and total soluble organic carbon (TSOC) in fecal samples **(C)** during run-in (week 1) and subsequent 3-week experimental phase.

**Figure 4:**
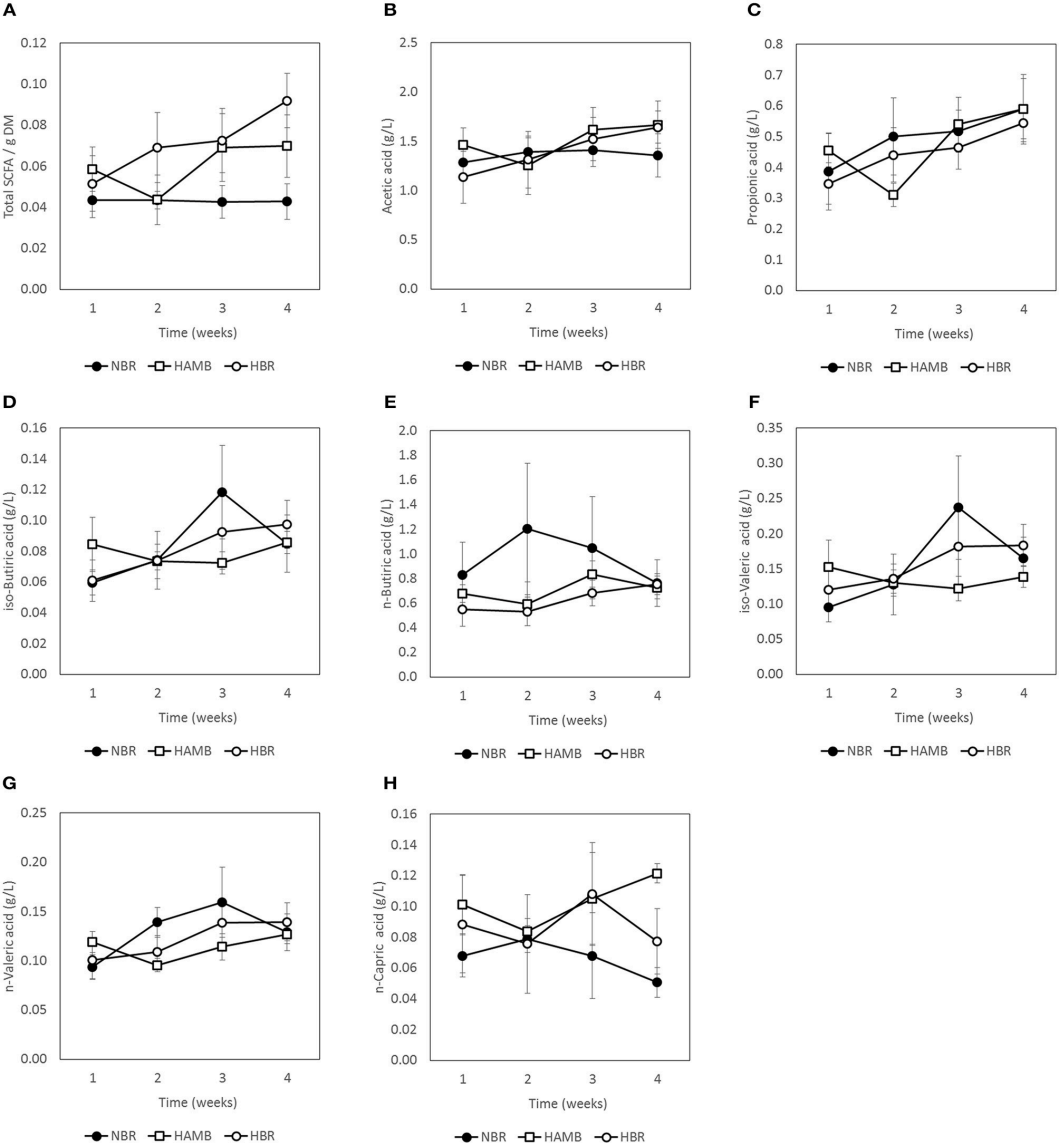
**Changes in concentration of C1–C6 short chain fatty acids (SCFA) present in fecal samples during run-in (week 1) and subsequent 3-week experimental phase: total SCFA (A)**, acetic acid **(B)**, propionic acid **(C)**, iso-butyric acid **(D)**, n-butyric acid **(E)**, iso-valeric acid **(F)**, n-valeric acid **(G)**, and n-capric acid **(H)**.

**Figure 5:**
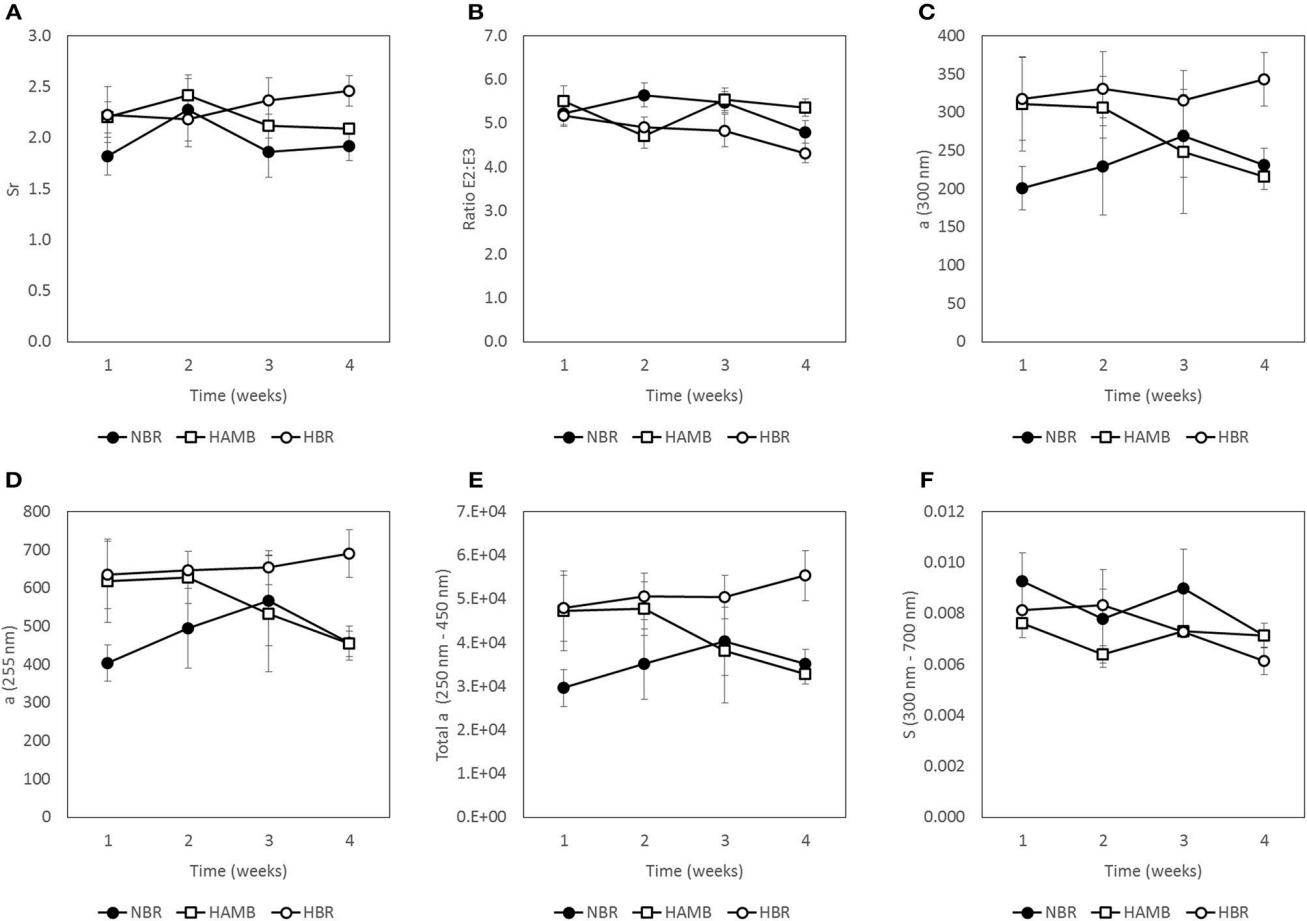
**Changes in molecular weight indices (MWI) of fecal samples during run-in (week 1) and subsequent 3-week experimental phase: ***Sr*** = ratio of absorption slopes between 275–295 and 350–400 nm slope (A)**, E2:E3 = ratio between absorption coefficients at 250 nm and at 365 nm **(B)**, a (300 nm) = Napierian absorption coefficient at 300 nm **(C)**, a (255 nm) = Napierian absorption coefficient at 255 nm **(D)**, Total a = integrated absorbance between 250 nm and 450 nm **(E)** and S = spectral slope of absorbance from 300 to 700 nm **(F)**.

No significant changes (*p* = 0.19) were detected between experimental variants in a number of measured gut metabolites and physicochemical parameters (Figures 3–5; Figure S3) despite the observed changes in stool consistency levels based on BSS. Increased fecal pH in both hypoxic conditions (week 2) coincided with hypoxia-related hyperventilation and blood alkalosis. In addition, the concentrations and ratios of butyrate and next two most important SCFA acetate and propionate were also variable and were not associated neither with time, inactivity or hypoxia, but showing response to differences in ingested food composition.

### Diversity and abundance of butyrate producing microbial community was not influenced by bedrest and hypoxia

**Figure 6:**
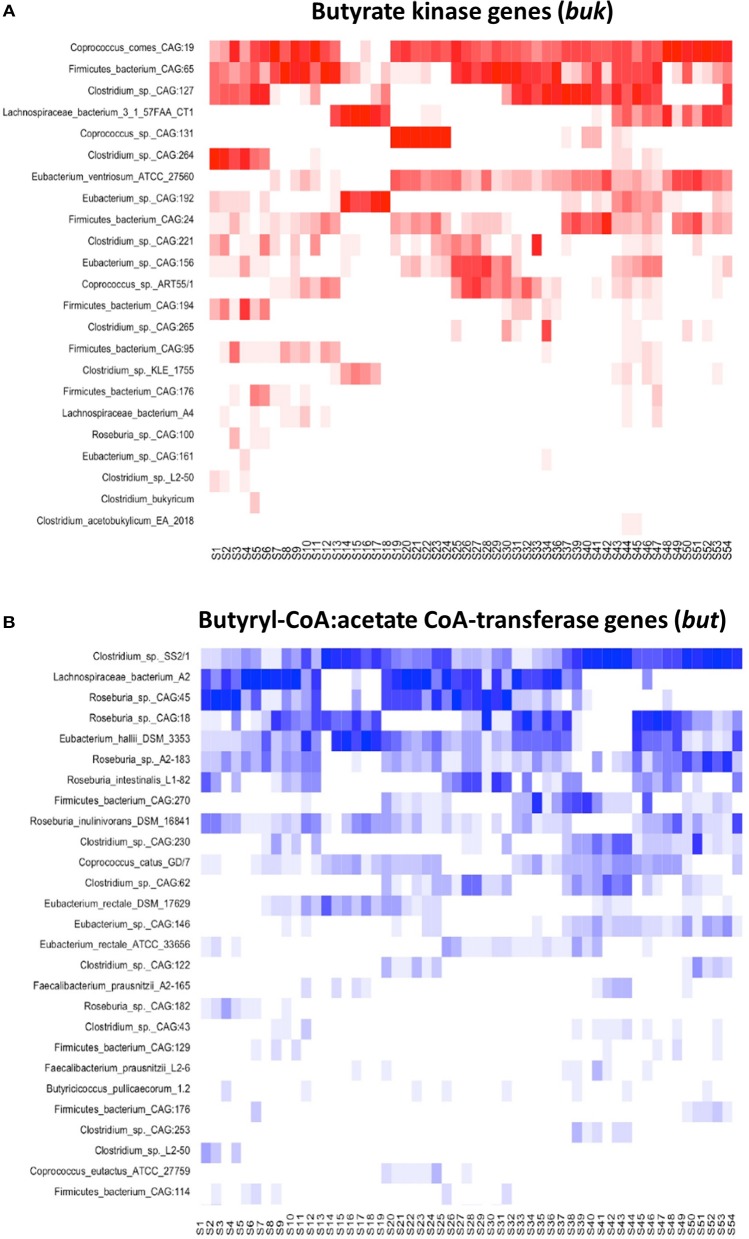
**A schematic representation of closest matches of ***buk*** (A)** and *but* **(B)** gene sequences describing butyrate producing communities in 54 PlanHab samples.

**Figure 7:**
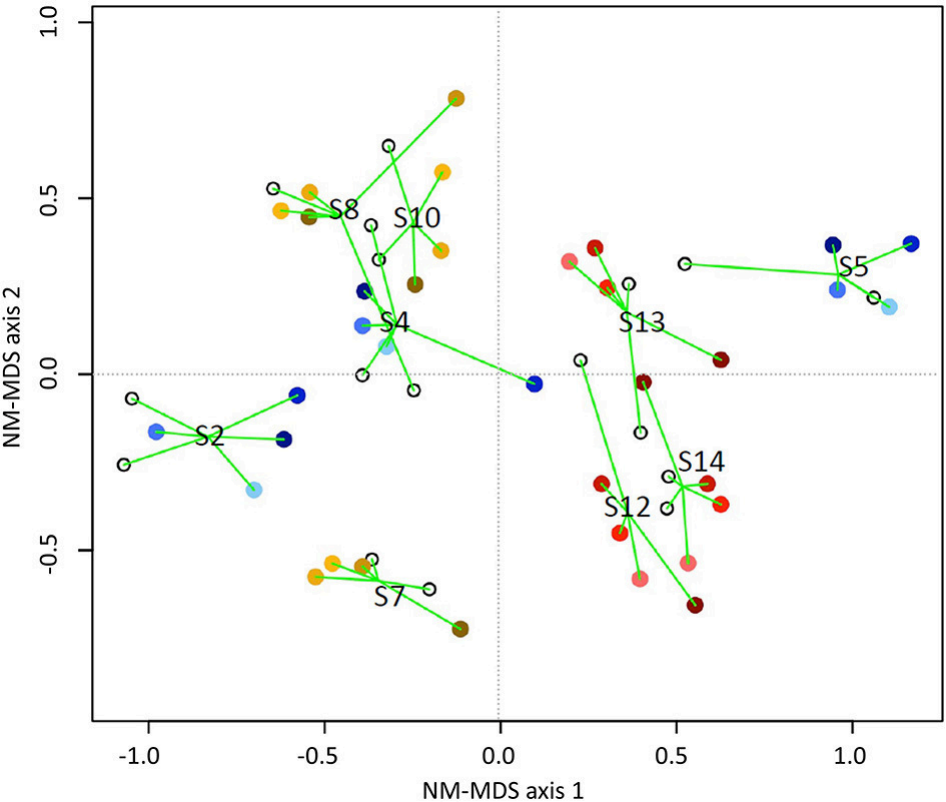
**NM-MDS ordination showing a host-specific grouping of butyrate communities based on combined ***but*** and ***buk*** gene datasets for all participants in experimental variants: NBR (blue), HBR (red), HAmb (yellow)**. The time spent in experiment is designated by darker colors. Stress_*buk*+*but*_ = 0.18. Only OTUs represented by at least 50 sequences were used in analyses. The numbers designate sample groupings according to participants.

Detailed sequence analysis of *but* and *buk* genes also showed that butyrate producing microbial communities were comparatively diverse in all experimental variants (Figures 6, 7). There was no significant difference in α-diversity estimates (*p* > 0.39) of *but* and *buk* genes between BDC and experimental time points, suggesting no detectable effect of the 21 day hypoxia and inactivity on the structure and diversity of butyrate microbial communities (Tables S3, S4).

**Figure 8:**
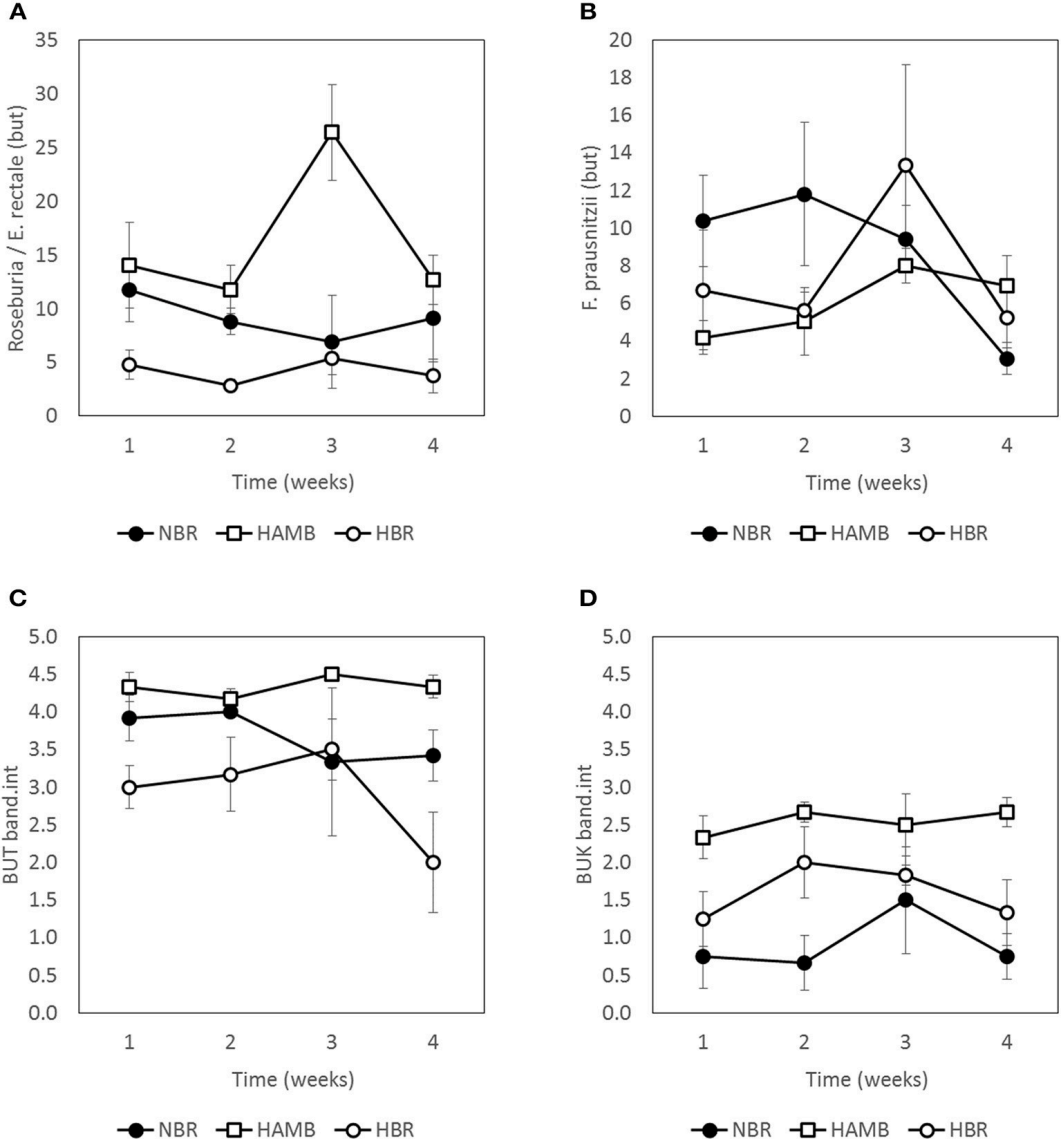
**Abundance of butyrate producing community members as estimated by two approaches, qPCR (A,B)**, and band intensities **(C,D)** during run-in (week 1) and subsequent 3-week experimental phase. Units are scaled to represent % of total bacterial microbial community **(A,B)**. Band intensities of PCR products **(C,D)** were categorized according to band intensity classes (0–5) (Vital et al., 2015).

Based on band intensity the more abundantly represented butyrate producing community harbored *but* genes, however, the abundance of *but* and *buk* genes was rather dynamic and within the same range in all samples (Figure 8). This is in line with the observation that the concentration of butyrate or other SCFA in samples did not change significantly over time within and between experimental variants.

The abundance of *Roseburia/E. rectale* and *F. prausnitzii* based on *but* gene qPCR analysis was comparable through the course of experiments, with the exception of week 3 of HAmb when the highest levels were transiently detected. The effects of experimental variant and time were not significant in two-way NP-MANOVA (*p* > 0.46) after the correction for multiple comparisons.

The observed fluctuations in abundance of butyrate producing community were correlated to menu composition (not shown). Butyrate producing community responded to fluctuations in dietary fiber content within the expected limits exhibiting its healthy physiological status.

### Correlative analysis

To determine the association between the data matrices recorded in this study (metabolites, immunological markers, experimental setup, diet; Table S1) and OTUs of butyrate producing communities, variation partitioning was conducted. A step-down procedure identified a smaller subset of variables out of all recorded metadata (*n* = 167) (Table S5) that were significantly associated with dispersion of butyrate producing microbial communities. A subset of all metabolic and immunological variables (butyrate, isoValerate, acetate, total SCFA, BA, EDN, TSOC, nCapreate, A1AT), experimental data [participant characteristics (age, height, BMI), activity level, experimental variant] as well as diet related parameters [histidine, Chloride ions, rare bacterial OTUs, ingested fat and water, sucrose, pantothenic acid (vitamin B5)] were ranked in descending order of importance within each, respectively (Table S5).

In general, variation in butyrate producing microbial community structure was significantly explained by experimental setup (13.4%), experimentally structured metabolites (12.8%), gut metabolite content and levels of immunological markers (11.9%). Despite the introduction of numerous variables into analyses (*n* = 167) many metadata variables were found to be correlated and hence were removed as redundant during the step-down procedure and excluded from further analyses. Consequently, 61.9% of variation in butyrate producing microbial community data remained unexplained and attributed to a combination of experimental and unknown, but real sources of variation, next to random noise.

## Discussion

### Changes in human physiology within PlanHab

The real-time 21 day exposure of healthy participants to three different settings induced significant adaptations in human physiology. Systemic hypoxia was associated with ≈10% reduction of SpO_2_ in HAmb and HBR (Keramidas et al., 2016). Combination of hypoxia and inactivity (HBR) resulted in general signs of congestion such as 10% increased mean arterial pressure, 15% increased heart rate, three times larger decrease in plasma volume and concomitant 3-fold increase in mature blood cells, hemoglobin and hematocrit concentrations in comparison to either NBR or HAmb. It has to be noted that systemic hypoxia could have thus also aggravated the inactivity-induced local tissue hypoxia in the HBR. General fatigue, tiredness, tension, recovery delay, negative affective responses, induced participativeness (i.e., extent of induced willingness to join in an activity) were also largest in HBR, followed by NBR, whereas almost undetectable at HAmb (Stavrou et al., 2016). Increased postprandial glucose concentration and reduced insulin sensitivity were reported in both inactive variants NBR and HBR (Simpson et al., 2016), suggesting the onset of obesity-related symptoms. At the same time, HAmb exhibited numerous positive attributes of human physiology such as decreased fasting insulin concentration, retained insulin sensitivity, unchanged postprandial serum insulin concentration and increased fasting fat oxidation rate, c-peptide response, c-peptide/insulin ratio (fed or fasted). Lastly, the negative effects observed in human physiology were reported to be dose dependent on the extent of time each participant was subjected to inactivity (NBR) or inactivity and hypoxia (HBR) (Simpson et al., 2016).

Exercise apparently influenced food preference to some extent as the amounts of ingested iron, proteins, fat and Ca^2+^ were significantly higher in HAmb (9, 10, 6, and 7.5%, respectively) in comparison to HBR or NBR (Debevec et al., 2014), similar to Clarke et al. (2014) observations for professional athletes. HAmb members also exhibited lower blood glucose, insulin sensitivity, reduced body fat, postprandial glucose, fasting serum total cholesterol, and lipoprotein cholesterol (Simpson et al., 2016) next to reduced inflammation markers (this study; Figure 2). Moderate activity in HAmb effectively precluded the development of intestinal inflammation and constipation despite the concomitant negative effects of systemic hypoxia coupled to inactivity as observed in HBR. As especially the benefits of exercise, that are well established for cardiovascular and metabolic health, extend to other organ systems including the gut (Cronin et al., 2016), the lack of exercise also reached and affected the gut on a short time scale of 21 days (NBR) and was additionally aggravated by systemic hypoxia (HBR), as shown in this study.

Tissue (NBR) and combination of tissue and systemic hypoxia (HBR) increased inflammatory responses in inactive variants were recently linked to central inflammatory mediators nuclear factor kappa B (NF-kB) and hypoxia inducible factor 1 (HIF-1) (Glover et al., 2016; Zeitouni et al., 2016) that modulate cell transcription, shape nutritional-immunity status of the gut and induce the release of reactive oxygen and nitrogen species (Faber and Bäumler, 2014). These reactive chemical species can act as additional electron acceptors for microbes, creating new niches for facultatively anaerobic bacteria and imposing a new selective force on microbial growth, their activity patterns and microbial cross-talk. From this it follows that the observed systemic inflammation levels in NBR and HBR were not produced by direct external microbial infection as the HAmb participants would become infected as well within the same experimental facility, receiving the same food and drinks, microbial burden, and aerosols. This points to one important link between exercise and constipation (i.e., decreased gut motility, Figure 1) for the pathologies in NBR and HBR observed in this study and apparent increase in intestinal anoxic ischemia (Haglund, 1994; Glover et al., 2016). The positive effects of exercise for reducing the negative effects of tissue hypoxia observed in NBR and exacerbated by systemic hypoxia in HBR were confirmed as even passive resistive vibration exercise decreased systemic inflammation observed in 60-day normoxic bedrest (Hoff et al., 2015).

### System resilience, increased stool consistency, and prolonged retention times

PlanHab experimental setup was tuned for 28 days in total to minimize the effects of factors, that have been identified as those contributing to changes in microbial community structure: host lifestyle and diet (David et al., 2014a; Conlon and Bird, 2015); diurnal oscillations (Thaiss et al., 2014); exercise (Clarke et al., 2014) and development (Voreades et al., 2014).

Contrary to the observed rapid and reproducible changes in human gut microbiome in response to an abrupt change from plant-based to animal-based diet and back (David et al., 2014b), the diet supplied to participants in this study was balanced to provide feeding habit continuation and resembled the ordinary menu composition of participants in their daily life. Differences in cyclic circadian oscillations, known to induce transkingdom control of microbiota diurnal oscillations that promote metabolic homeostasis (Thaiss et al., 2014) were minimized through the adoption of a 16/8 daylight regime in this study (Debevec et al., 2014). In addition, the timing of food delivery was kept constant in order to minimize variation in flow-rate of material through the gut that was shown previously to influence microbial and host metabolic activities (Gilbert and Alverdy, 2016).

In essence, strict cessation of exercise in NBR and HBR induced significant changes in human physiology, food preference (Debevec et al., 2014), deconditioning of several vital subsystems, development of systemic inflammation and insulin resistance (Simpson et al., 2016), but apparently little congruent changes in butyrate producing microbiome as observed in this study.

The growth rate and ability to modulate microbial physiology and adherence to the gut mucosal layer are most likely the key structuring parameters of microbial communities (Gilbert and Alverdy, 2016). A significant increase in transit time would most probably increase competitiveness of k-strategists favoring more stable ecological niches increasing the extent of their adaptive activities and metabolic fingerprints resulting in alteration of microbial community structure. Increased stool consistency measured according to BSS was recently associated with increased α-diversity in females (Vandeputte et al., 2016). However, later and larger studies observed strong associations of stool consistency with microbiota composition, but could not confirm association of BSS with microbial richness (α-diversity) or gender differences (Tigchelaar et al., 2015). Despite the increased stool consistency and prolonged retention times observed in NBR and HBR variants (Figure 1), no significant difference in butyrate concentration or α-diversity of butyrate producing communities (*but* and *buk*) was observed between experimental variants.

The lack of directed changes in butyrate producing communities also suggests that the microbial intestinal system exhibited possibly an evolutionary resilience toward short-term modifications due to inactivity or hypoxia in this study. However, the same microbial components might be responding to tissue hypoxia, inactivity and additional systemic hypoxia derived inflammation responses by adjusting their metabolic activities, supporting the development of host pathologies on the long run (i.e., >21 days; Faber and Bäumler, 2014). This finding is at least in part supported by recent findings where lack of change in response to bedrest or inactivity was observed for highly personalized structures of general bacterial communities in the same samples (Šket et al., 2015). In addition, upon cessation of the 21 day experimental period (NBR or HBR), the wash-out of acutely developed negative physiological symptoms lasted approximately 14 days (Debevec et al., 2014).

### Intestinal transport and abrasions

There are potentially numerous other parameters that have so far been completely overlooked in analyses of microbiome such as fasting and postprandial gastrointestinal mobility that regulates transport of chime, the intervals within interdigestive periods [(i) quiescence, (ii) irregular contractions, (iii) migrating motor complexes (MMC)]. In essence, the contractile patterns of small intestine that propagate toward the colon are caused by the enteric nervous system, the interstitial cells of Cajal that generate slow waves of smooth muscle contraction. These determine the maximal frequency of intestinal contractions, generating far spreading and fast peristaltic waves at the proximal small intestine that become shorter and slower toward the distal gut, and contribute to the different transit rates along the intestine (Ehrlein and Schemann, 2005; Johnson et al., 2012). The transport of chime decreases along the gut in the same proportion as the volume of luminal content declines by absorption of nutrients and water. In addition, with increasing filling of distal intestinal segments the motility of the proximal intestine is inhibited, the number of peristaltic waves decreases while the number of stationary segmenting contractions increases. The number and the length of peristaltic waves, i.e., circular constrictions propagating aborally, determine chime transport. Stationary or segmenting contractions, isolated at a single site without spatio temporal pattern, are pushing, mixing and separating chime into segments, possibly inflicting intestinal abrasions during prolonged and increasing constipation. The derived abrasions are highly amenable for further microbial colonization leading to increased local inflammation on the long run. This is especially true for locations with a thin or modified mucus layer (e.g., small intestine, abrasions, reduced mucus thickness, increased porosity and modified mucus glycosylation pattern; Glover et al., 2016).

### Chime energetic density and microbial colonization

Nutrients entering the distal small intestine induce feed-back regulation that reduces gastric emptying to ensure increased yields from high energetic density chime but also slows down the flow rate of chime along the gut (Ehrlein and Schemann, 2005). During the short-lasting increase in energetic density of the chime the digestive period of the gut is only transiently prolonged, as observed in NBR and HBR. However, during long-lasting increases in energy intake (high-density food), the absorption capacity of the gut is enhanced by processes of intestinal tissue adaptation, a characteristic of obesity-related syndromes.

Under the conditions of decreasing flow rate, i.e., increasing constipation, the basic MMC contractions clean the small intestine from chime residues and host excreta in order to prevent bacterial overgrowth. Waves push the chime a few centimeters aborally followed by partial back-flow during the period of relaxation. This cleaning is supported by enhanced secretion of gastric and pancreatic juices and BA immediately before MMC contraction (Ehrlein and Schemann, 2005).

Fiber and water are essential at this step as they maintain the bulk and substitute the pool of otherwise readily available soluble compounds making them less accessible to microbes, decreasing thus the energy density per surface area of the chime. The increased availability of simple organic molecules to fast growing microbes in absence of fiber can provide competitive advantage to those microorganisms in slowly moving small intestine that are capable of biofilm formation through attachment and colonization of otherwise largely microbiota-free small intestine surface. Rather subtle changes in microbial colonization during biofilm formation effectively lead to modified metabolic activity patterns via quorum sensing behavior. Microbial signals are effectively amplified by the 15–20 times larger surface area of small intestine villi and microvilli in comparison to large intestine where fiber fermentation has the potential to effectively provide stable nieche characteristics (Helander and Fändriks, 2014).

### BA, EDN, and microbial overgrowth

Increased EDN levels in NBR and HBR pointed to progressive development of intestinal inflammation. The lack of change in other parameters of the colon such as butyrate producing community, SCFA concentrations, epithelial and mucosal integrity and permeability recorded in this study are in line with reports that physiologic hypoxia predominates in colon (Glover et al., 2016). Hence its health status and oxygenation are under the control of SCFA, not postprandial hyperaemia as was observed in small intestine (Zheng et al., 2015). This indicates that the on-site inflammation observed through EDN is probably taking place in the small intestine that is more susceptible to microbial overgrowth under the conditions of slowed chime transport due to high-energy (Western) diet intake, physical inactivity and associated hypoxia, increasing constipation and associated fluctuations in oxygen availability.

Increased BA levels observed in NBR and HBR originate from increased liver BA excretion into the small intestine in an attempt of the host to increase gut motility and decrease small-intestine overgrowth by commensal microbiota. In addition, the facts that BA are known to be potent vasodilators (Ward et al., 2014; Zheng et al., 2015) and play a key role in intestinal hyperaemia point again to the small intestine as the starting location of tissue hypoxia under physical inactivity and consequent associated inflammation in response to microbial overgrowth activities.

### Limitations and future directions

A few limitations and concepts of this study need to be considered as well. Firstly, the sample size in this study was relatively small, but well within the limits of recent detailed studies (David et al., 2014a,b; Schloss et al., 2014; Thaiss et al., 2014). Second, the limited statistical power and accompanying potential for type-II error was at least partly alleviated by the fact that the test participant population was prescreened for healthy young males according to SOP used by ESA/NASA and the study executed according to SOP for bedrest studies. Nevertheless, a larger study sampling random population longitudinally as participants experience variations in intestinal transit times might be able to find additional and more significant effects. Third, many of the initially measured variables were found to be correlated and hence did not contribute to the partitioning of variability in butyrate producing microbial communities. From an analytical perspective these results suggest that measurements at different scales using the current set of variables and/or inclusion of additional variables would be needed in future analyses in order to delineate the extent of random noise. Last, the current study represents a proof of principle that numerous variables are in fact implicated into development of systemic inflammation and that transdomain systems biology research on the same participants within the same experiments deploying a diversity of research objectives, subsystem monitoring and tools (physiology, body traits, body composition, immunology, psychology, neuroendocrine, nutrients, appetite, microbial communities, constipation, water intake, temperature control, sleep architecture, antioxidant defenses) is feasible and could be integrated into future studies adopting multiomics approaches. Future experiments utilizing conditions without hypoxia and inactivity may be a good addition to understand potential interaction between tissue-hypoxia and exercise over prolonged monitoring periods, e.g., >60 days.

## Conclusions

In conclusion, this study provides evidence that increasing constipation and gut inflammation appear shortly after onset of physical inactivity (lack of physical exercise in NBR) that were potentially exacerbated by systemic hypoxia (during HBR) and significantly alleviated by exercise, despite hypoxia (HAmb). Gut permeability, concentration of crucial intestinal metabolites, structure and abundance of butyrate producing microbial communities in the colon did not change significantly suggesting that the intestinal system apparently exhibited a resilience toward short-term modifications in host exercise levels or systemic hypoxia. Progressive constipation (decreased intestinal motility) and increased local inflammation markers suggest that changes in microbial colonization and metabolism were taking place at the location of small intestine.

## Ethics statement

This study was carried out in accordance with the recommendations of European Space Agency's standardization plan for bed rest studies and National Committee for Medical Ethics at the Ministry of Health of the Republic of Slovenia with written informed consent from all subjects. All subjects gave written informed consent in accordance with the Declaration of Helsinki. The protocol was approved by the National Committee for Medical Ethics at the Ministry of Health of the Republic of Slovenia.

## Author contributions

Designed and executed PlanHab experiment: IM, OE, and TD. Provided concept for microbiome analysis: BS, MS, and JT. Collected samples: TD and BS. Designed *buk* and *but* qPCR and NGS: MV, JC, and JT. Conducted research on metabolites, inflammation markers, BSS and other data: BS, RS, and NT. Analyzed data: BS, RS, MV, and JC. Drafted manuscript: BS. Provided ideas: BS, MS, JT, and MV. All authors contributed to the final version of the manuscript and authorized its final version.

## Funding

The study was funded by the European union programme FP7 (PlanHab project; Grant no. 284438), the European Space Agency (ESA) Programme for European Cooperating States (ESTEC/Contract No. 40001043721/11/NL/KML: Planetary Habitat Simulation), and the Slovene Research Agency (Contract no. L3-3654: Zero and reduced gravity simulation: The effect on the cardiovascular and musculoskeletal systems). RS was supported through Young Research Fellowship (SRA#37426), Slovenian Research Agency.

### Conflict of interest statement

The authors declare that the research was conducted in the absence of any commercial or financial relationships that could be construed as a potential conflict of interest.

## Abbreviations

a: Napierian absorption coefficient
A1AT: α_1_-antitrypsin
BA: Bile acids
BDC: Baseline data collection
BSS: Bristol stool scale
DOM: Dissolved organic matter
EDN: Eosinophil-derived neurotoxin
ESA: European Space Agency
F_i_O_2_: Fraction of inspired O_2_
HAmb: Normobaric hypoxic ambulatory confinement
HBR: Normobaric hypoxic bed rest
MMC: Migrating motor complexes
MW: Molecular weight
MWI: Molecular weight indices
NBR: Normobaric normoxic bed rest
NM-MDS: Non-metric multidimensional scaling
OTUs: Operational taxonomic units
P_i_O_2_: Partial pressure of inspired O_2_
qPCR: Quantitative PCR
SCFA: Concentration of C1–C6 short chain fatty acids
SpO_2_: Capillary oxyhemoglobin saturation
Sr: Ratio slopes between 275–295 and 350–400 nm slope
TSOC: Total soluble organic carbon.
