# Supplementary material for: Hypoxia and Inactivity Related Physiological Changes (Constipation, Inflammation) Are Not Reflected at the Level of Gut Metabolites and Butyrate Producing Microbial Community: The PlanHab Study
Source: Front Physiol. 2017 May 4;8:250. doi: 10.3389/fphys.2017.00250 (PMC5416748; doi:10.3389/fphys.2017.00250)
Supplement: Supplementary file 3 [file DataSheet3.pdf]

Electronic Supplementary Material

Hypoxia and inactivity related physiological changes (constipation, inflammation) are not reflected at the level of gut metabolites and butyrate producing microbial community: The PlanHab study

Robert Šket[1], Nicole Treichel[2], Tadej Debevec[3], Ola Eiken[4], Igor Mekjavic[3], Michael Schloter[2], Marius Vital[5], Jenna Chandler[5], James M. Tiedje[5], Boštjan Murovec[6], Zala Prevoršek[1], Blaž Stres[1,7]*

[1]Department of Animal Science, Biotechnical Faculty, University of Ljubljana, Ljubljana, Slovenia

[2]Research Unit for Comparative Microbiome Analysis, Helmholtz Zentrum München - German Research Center for Environmental Health, Neuherberg, Germany

[3]Department of Automation, Biocybernetics and Robotics, Jozef Stefan Institute, Ljubljana, Slovenia

[4]Department of Environmental Physiology, Swedish Aerospace Physiology Centre, Royal Institute of Technology, Stockholm, Sweden

[5]Michigan State University, Center for Microbial Ecology, East Lansing, Michigan, USA

[6]Laboratory for artificial sight and automation, Faculty of Electrical Sciences, University of Ljubljana, Ljubljana, Slovenia

[7] Center for clinical neurophysiology, Faculty of Medicine, University of Ljubljana, Ljubljana, Slovenia

*Corresponding author: Blaž Stres, Department of Animal Science, Biotechnical Faculty / Center for clinical neurophysiology, Faculty of Medicine, University of Ljubljana, Jamnikarjeva 101, 1000 Ljubljana, Slovenia.

Email: blaz.stres@bf.uni-lj.si; Tel: +386 3203 869

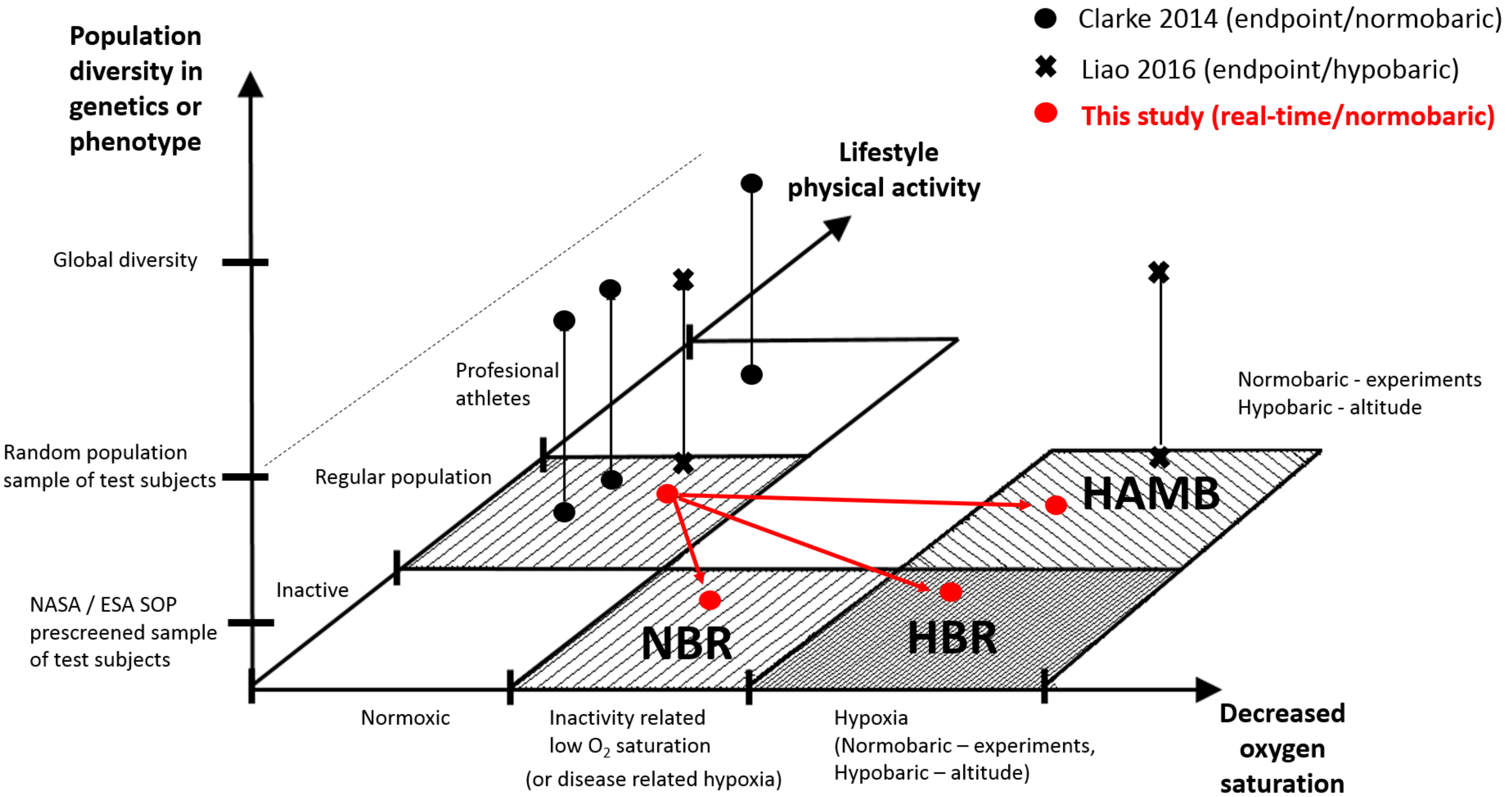

Figure S1: Human systems biology exploration space mapping exercise with oxygen saturation levels and human population diversity (Clarke et al., 2014; Debevec et al., 2014; Liao et al., 2016).

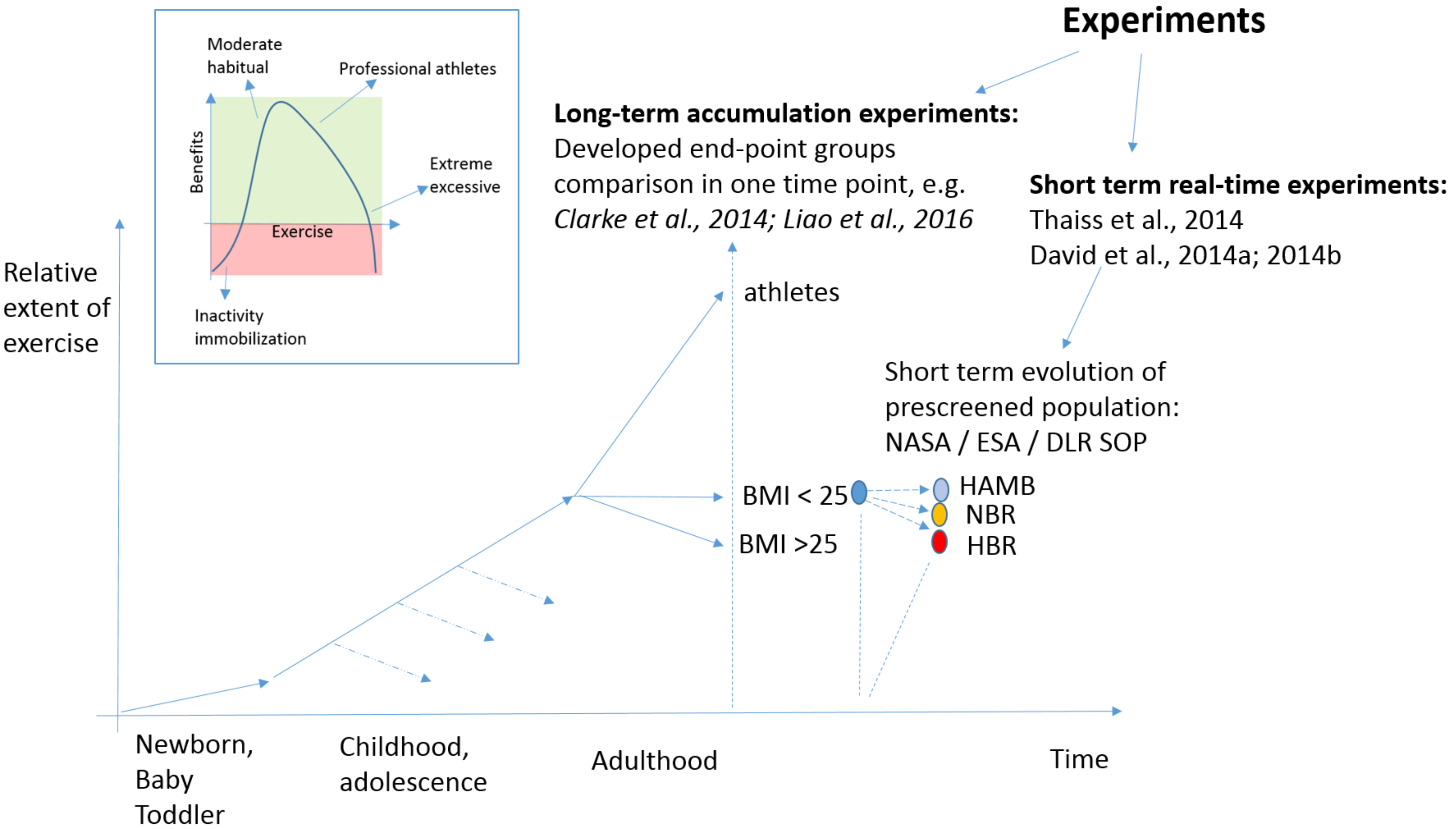

Figure S2: The difference between accumulation and short-term real-time experiments. The inset shows a tentative scheme of dose dependent benefits derived from various levels of exercise (Bermon et al., 2015; Cronin et al., 2016; Egan and Zierath, 2013; Ringseis et al., 2015).

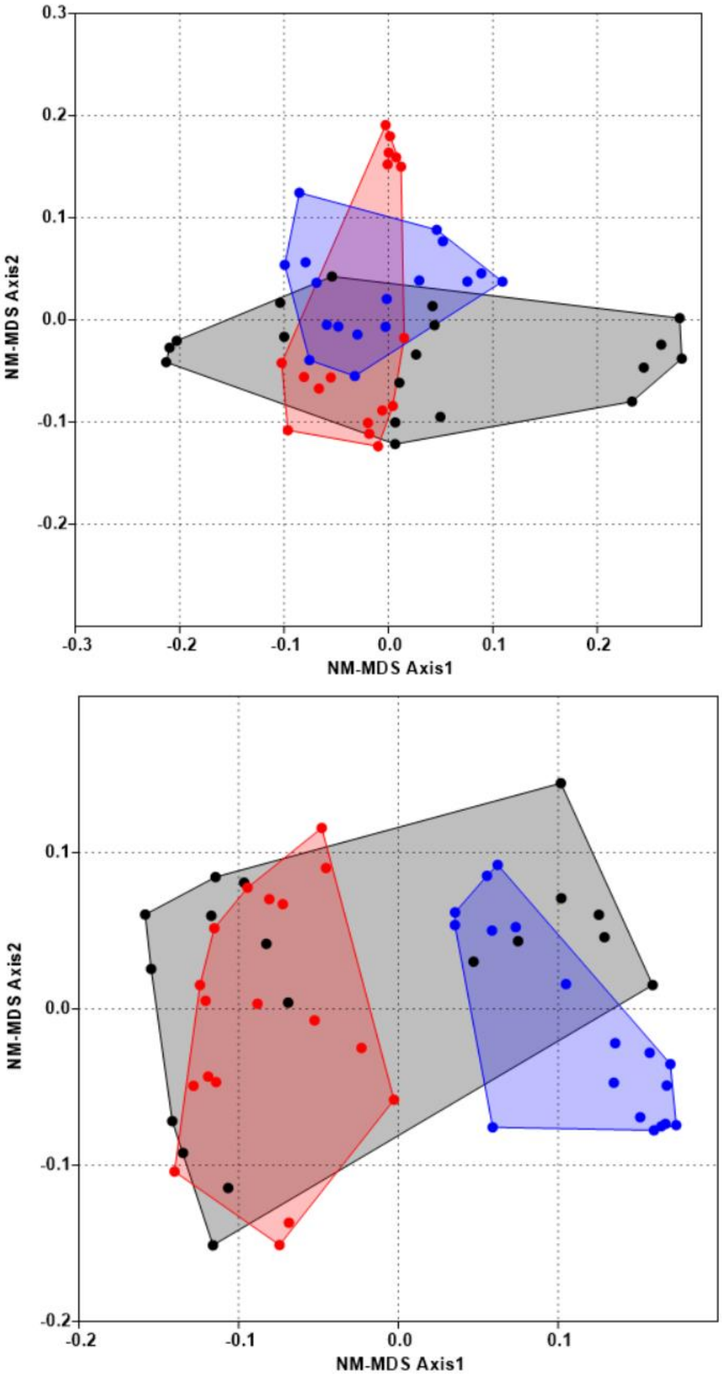

Figure S3: NM-MDS ordination of *buk* (A) and *but* (B) genes. NBR (black), HBR (red), HAmb (blue). $Stress_{buk}$ = 0.17, $Stress_{but}$ = 0.16.

Table S1: An overview of the three data matrices containing (i) experimental, (ii) diet, (iii) metabolites and immunological parameters used in variation partitioning of butyrate producing microbial community datasets in this study.

**Experiment**	**Diet**				**Metabolite and immuno**
Participant (n = 9)	aaeb - Branched-chain amino acids (g	f18_2tn - "no name" (g)	fsugar - Available sugars (g)	protan - Protein of animal origin (g)	pH
Hypoxia (n = 2)	aat - Total amino acids (g)	f18_3 - Linolenic fatty acid (g)	gals - Galactose (g)	protpl - Protein of plant origin (g)	reducing sugars - Colorimetric detection of reducing sugars content
Inactivity (n = 2)	ala - Alanine (g)	f18_3cn3 - "no name" (g)	gi - GI - glycemic index	ribf - Vitamin B2 (mg)	%water.feces - Water content in feces (%)
Sample (n = 54)	alc - Alcohol (g)	f18_3cn6 - "no name" (g)	gl - GL - glycemic load	se - Selenium (µg)	Acetic - Acetic acid (g/L)
Time in experiment(n = 6)	arg - Arginine (g)	f18_3n3 - "no name" (g)	gln - Glutamine (g)	ser - Serine (g)	Propionic - Propionic acid (g/L)
Experimental variant (n = 3)	asp - Aspartate (g)	f18_3n6 - "no name" (g)	glu - Glutamate (g)	sq - "no name"	isoBut - iso-Butiric acid (g/L)
Age (year)	biot - Vitamin B7 (µg)	f18_4n3 - "no name" (g)	glus - Glucose (g)	ssugar - Total simple sugars (g)	nBut - n-Butiric acid (g/L)
Height (cm)	ca - Calcium (mg)	f20_2n6 - "no name" (g)	gly - Glycine (g)	starch - Starch (g)	isoVal - iso-Valeric acid (g/L)
Body mass (kg)	carta - alpha-Carotene (µg)	f20_3n6 - "no name" (g)	his - Histidine (g)	sucs - Sucrose (g)	nVal - n-Valeric acid (g/L)
BMI - Body mass index	cartb - beta-Carotene(µg)	f20_4n6 - "no name" (g)	id - Identifier	sugar - Total sugars (g)	nCapric - n-Capric acid (g/L)
	cho - Total carbohydrates (g)	f20_5n3 - "no name" (g)	ile - Isoleucine (g)	thia - Vitamin B1 (mg)	totSCFA - Total short chain fatty acids (g/L)
	chorl - Cholesterol (mg)	f22_4n6 - "no name" (g)	k - Potassium (mg)	thr - Threonine (g)	TSOC - Total soluble organic compounds per gram of dry matter in feces
	chot - Total carbohydrates (g)	f22_5n3 - "no name" (g)	kcal - Kilocalories	trp - Tryptophan (g)	BA - Bile acids (fold)
	chot_p - Total carbohydrates (%)	f22_6n3 - Docosahexaenoic fatty acid (g)	lacs - Lactose (g)	tyr - Tyrosine (g)	zonulin - Zonulin (fold)
	cld - Chlorine (mg)	fams - Monounsaturated fatty acids (g)	leu - Leucine (g)	val - Valine (g)	A1AT - α- 1 anti-trypsin (fold)
	color - Color	fapu - Total polyunsaturated fatty acids(g)	lys - Lysine (g)	vita - Vitamin A (µg)	EDN - Eosinophile derived neurotoxin (fold)
	cr - Chromium (µg)	fapun3 - Total omega-3 fatty acids (g)	mals - Maltose (g)	vitb12 - Vitamin B12 (µg)	BSS - Bristol stool scale
	cu - Copper (µg)	fapun6 - Total omega-6 fatty acids (g)	met - Methionine	vitb6 - Vitamin B6 (mg)	retention time - Time between particular defecations (days)
	cyste - Cysteine (g)	fasat - Saturated fatty acids (g)	mg - Magnesium (mg)	vitc - Vitamin C (mg)	defecation frequency - Number of defecations during experiments
	edible - "no name"	fat - Total fat (g)	mn - Manganese (µg)	vitd - Vitamin D (µg)	Sr - Ratio of absorption slopes between 275-295 nm slope and 350-400 nm slope
	enera - Energy (kcal)	fat_p - Total fat (%)	mo - Molybdenum (µg)	vite - Vitamin E (mg)	E2.E3 - Ratio between absorption coefficients at 250 nm and at 365 nm
	f14_0 - Myristic fatty acid (g)	fd - Fluoride (µg)	na - Sodium (mg)	vitk - Vitamin K(µg)	a255 - Absorption coefficient at 255 nm
	f16_0 - Palmitic fatty acid (g)	fe - Iron (mg)	nacl - Sodium chloride (g)	water - Water (g)	a.300 - Absorption coefficient at 300 nm
	f18_0 - Stearic fatty acid (g)	fibc - Crude fiber (g)	nia - Vitamin B3 (µg)	zn - Zinc (mg)	Total.a.250.450 - Integrated absorbance between 250 nm and 450 nm
	f18_1cn9 - Oleic fatty acid (g)	fiber.energy.kcal - Total fiber energy yield (kcal)	p - Phosphorus (mg)		S.300.700 - Spectral slope of absorbance from 300 nm to 700 nm
	f18_2 - Linoleic fatty acid(g)	fibins - Insoluble fiber (g)	pantac - Vitamin B5 (mg)		
	f18_2cn6 - "no name" (g)	fibsol - Soluble fiber (g)	phe - Phenylalanine (mg)		
	f18_2con - "no name" (g)	fibt - Total fiber (g)	pro - Proline (g)		
	f18_2ct - "no name" (g)	fol - Folic acid (µg)	prot - Total protein (g)		
	f18_2iso - "no name" (g)	frus - Fructose (g)	prot_p - Total protein (%)		

Table S2: Comparison of observed trends in four immunological markers after the completion of experiments relative to BDC values. NBR- normobaric normoxic bedrest, HBR – normobaric hypoxic bedrest, HAmb – normobaric hypoxic ambulation. Please see details in extended captions.

	mode of action	**NBR**	**HBR**	**HAmb**	*Interpretation*
**Zonulin**[1]	increases tight junction degradation and permeability of epithelia	0	0	0	*low is good*
**A1AT**[2]	signals damaged mucosal integrity and increased permeability	0	0	0	*high is good*
**EDN**[3]	signals location of inflammation, site of tissue damage	+>200%	+>300%	0	*low is good*
**BA**[4]	marker of FFA metabolism, precursor for secondary BA, exhibits toxicity for bacteria, increases proinflamatory cytokines	+>200%	+>150%	0	*low is good*

[1]**Zonulin** binds to a specific receptor on the surface of intestinal epithelia and triggers a cascade of biochemical events which induces tight junction disassembly and a subsequent permeability increase of the intestinal epithelia, allowing some substances to pass through and activate immune reactions. It is suggested that increased levels of zonulin are a contributing factor to the development of intestinal disorders and other autoimmune disorders.

[2]**α_1-antitrypsin** represents the majority of serine protease inhibitors and protects tissues from protease damages during inflammation. The protein is synthesized primarily in the liver but also to a small extent in intestinal macrophages, monocytes, and intestinal epithelial cells. Since α1-antitrypsin is relatively resistant against enzymatic digestion, the secreted amount in stool reflects the internal concentration of the protein. An elevated α_1-antitrypsin stool concentration is therefore a widely recognized marker for intestinal protein loss and for an increased mucosal permeability. Intestinal protein loss is a serious consequence of various systemic or local gastrointestinal diseases (e. g. allergies, chronic inflammation, malignancies). These pathologies damage the mucosal integrity and/or cause lymphostasis, thereby leading to an increased transfer of plasma proteins into the bowel lumen.

[3]**EDN (eosinophil-derived neurotoxin**, eosinophil protein x, EPX), a cationic glycoprotein, which is released by activated eosinophils, has strong cytotoxic characteristics and plays a significant role in the prevention of virus infections. It is released by the eosinophil granules in places where eosinophils are mainly found: in the skin, lungs, urogenital and gastrointestinal tract, that is, in the organs acting as an entry point for pathogens. The accumulation of EDN in the intestine is associated with inflammation and tissue damage. Measuring of EDN in stool can serve as an objective parameter for a current clinical or sub-clinical chronic inflammation located in the gastrointestinal area. In the case of Colitis ulcerosa and Crohn's disease, the EDN measurement enables the evaluation of a disease's activity and the prediction of a relapse.

[4]**Bile acids** are produced in the liver as end-products of cholesterol metabolism. Together with other components of the liver bile, such as cholesterol, bilirubin, phospholipids and proteins, bile acids are secreted into the duodenum. Important functions of bile acids are the excretion of cholesterol, absorption of fatty acids and fat-soluble vitamins in the small intestine as well as stimulation of intestinal motility. The majority of the secreted bile acids are reabsorbed in the terminal ileum and returned to the liver via the portal venous system for eventual recirculation in a process known as enterohepatic circulation; only a small proportion (3-5 %) are excreted into the feces. If the enterohepatic recycling of bile acids fails, excess amounts of bile acids enter the colon and are lost with the feces; this condition is called bile acid malabsorption.

Table S3: Supplementary file containing *buk* diversity estimates.

Table S4: Supplementary file containing *but* diversity estimates.

Table S5: Overview of selected variables after step-down procedure to be used in variation partitioning.

**Experiment**	Var.N	LambdaA	P	F
Age	8	0.11	0.002	6.06
Height	9	0.08	0.002	4.76
activity	3	0.07	0.002	4.61
Exp.variant	1	0.05	0.002	3.23
individual	7	0.08	0.002	6.55
BMI	11	0.05	0.002	4.03
Total:		**0.44**		

**Metabolite+Imuno**	Var.N	LambdaA	P	F
nButyrate	18	0.08	0.002	4.09
isoValerate	21	0.06	0.002	3.72
Acetate	8	0.04	0.002	2.44
Total SCFA	29	0.03	0.002	2.36
Bile Acids	36	0.04	0.004	2.36
EDN	37	0.03	0.002	2.22
TSOC	33	0.04	0.004	2.11
nCapreate	27	0.02	0.008	1.96
A1AT	35	0.03	0.006	1.95
Total:		**0.42**		

**Diet**	Var.N	LambdaA	P	F
histidin	70	0.04	0.008	2.06
Chloride ion	18	0.04	0.008	1.88
Rare bact. OTUs	116	0.03	0.008	1.88
Ingested fat	3	0.03	0.008	1.91
Ingested water	112	0.03	0.008	1.6
Sucrose	98	0.03	0.008	1.72
Vitamin B5	86	0.03	0.008	1.47
Total:		**0.23**		
